# Diet Rich in Lard Promotes a Metabolic Environment Favorable to *Trypanosoma cruzi* Growth

**DOI:** 10.3389/fcvm.2021.667580

**Published:** 2021-05-25

**Authors:** Débora Maria Soares de Souza, Maria Cláudia Silva, Silvia Elvira Barros Farias, Ana Paula de J. Menezes, Cristiane Maria Milanezi, Karine de P. Lúcio, Nívia Carolina N. Paiva, Paula Melo de Abreu, Daniela Caldeira Costa, Kelerson Mauro de Castro Pinto, Guilherme de Paula Costa, João Santana Silva, André Talvani

**Affiliations:** ^1^Laboratory of Immunobiology of Inflammation, Department of Biological Sciences, Federal University of Ouro Preto, Ouro Preto, Brazil; ^2^Biological Science Post-graduate Program, Federal University of Ouro Preto, Ouro Preto, Brazil; ^3^Health and Nutrition Post-graduate Program, Federal University of Ouro Preto, Ouro Preto, Brazil; ^4^Department of Biochemistry and Immunology, Ribeirão Preto Medical School, University of São Paulo, São Paulo, Brazil; ^5^Laboratory of Metabolic Biochemistry, Department of Biological Sciences, Federal University of Ouro Preto, Ouro Preto, Brazil; ^6^Center of Research in Biological Sciences, Federal University of Ouro Preto, Ouro Preto, Brazil; ^7^School of Physical Education, Federal University of Ouro Preto, Ouro Preto, Brazil; ^8^Fiocruz-Bi-Institutional Translational Medicine Plataform, Ribeirão Preto Medical School, University of São Paulo, São Paulo, Brazil; ^9^Health Science, Infectology and Tropical Medicine Post-graduate Program, Federal University of Minas Gerais, Belo Horizonte, Brazil

**Keywords:** inflammation, *Trypanosoma cruzi*, saturated fatty acids, monounsaturated fatty acids, adipose tissue, cardiac tissue

## Abstract

**Background:** *Trypanosoma cruzi* is a protozoan parasite that causes Chagas disease and affects 6–7 million people mainly in Latin America and worldwide. Here, we investigated the effects of hyperlipidic diets, mainly composed of olive oil or lard on experimental *T. cruzi* infection. C57BL/6 mice were fed two different dietary types in which the main sources of fatty acids were either monounsaturated (olive oil diet) or saturated (lard diet).

**Methods:** After 60 days on the diet, mice were infected with 50 trypomastigote forms of *T. cruzi* Colombian strain. We evaluated the systemic and tissue parasitism, tissue inflammation, and the redox status of mice after 30 days of infection.

**Results:** Lipid levels in the liver of mice fed with the lard diet increased compared with that of the mice fed with olive oil or normolipidic diets. The lard diet group presented with an increased parasitic load in the heart and adipose tissues following infection as well as an increased expression of *Tlr2* and *Tlr9* in the heart. However, no changes were seen in the survival rates across the dietary groups. Infected mice receiving all diets presented comparable levels of recruited inflammatory cells at 30 days post-infection but, at this time, we observed lard diet inducing an overproduction of CCL2 in the cardiac tissue and its inhibition in the adipose tissue. *T. cruzi* infection altered liver antioxidant levels in mice, with the lard diet group demonstrating decreased catalase (CAT) activity compared with that of other dietary groups.

**Conclusions:** Our data demonstrated that *T. cruzi* growth is more favorable on tissue of mice subjected to the lard diet. Our findings supported our hypothesis of a relationship between the source of dietary lipids and parasite-induced immunopathology.

## Introduction

The protozoan *Trypanosoma cruzi*, the etiological agent of Chagas' disease, affects 6–7 million people worldwide (1). The parasite triggers an intense tissue inflammatory response in the mammalian host (2, 3), which culminates in damage to cardiac cells. The progressive myocarditis is associated with high mortality and morbidity rates (4, 5).

In the absence of an effective pharmacological treatment against the *T. cruzi*, there has been a growing demand for methods that control the parasite replication and regulate the parasite induced immune response to minimize tissue damage in infected hosts (6). In this sense, it has been argued that the nutritional status and dietary quality might be of importance for the regulation of the host immune responses and in the progression of infection (7–9). Diets rich in saturated fatty acids have been linked to increasing adiposity and comorbidities such as diabetes, hypertension, atherosclerosis, as well as interfering in the immune response that favor the growth of *T. cruzi* (10, 11). Diets rich in monounsaturated and polyunsaturated fatty acids are beneficial to the body as they improve cardiac function and modulate the immune system (12, 13).

Considering that *T. cruzi* infection causes a chronic systemic and cardiac pattern of inflammatory response, and different lipids are present in the intra- and extracellular environmental of the parasites, the present study we aimed to investigate the interference of monounsaturated and saturated fatty acid diets in the course of *T. cruzi* infection related inflammation.

## Materials and Methods

### Ethical Approval

All the methodologies performed are in accordance with the standards of the National Council for Control of Animal Experimentation (CONCEA), and were previously approved by the Animal Research Ethics Committee of the Federal University of Ouro Preto (CEUA-UFOP), under the protocol number 36/2015. The experiments comply with the standards of animal research explicit in Law 11.749, of 2008, regulated by Decree No. 6.899, of July 15, 2009.

### Animals, Study Design, and Diets

C57BL/6 male mice aged 21-days were used. The animals were divided into groups according to the composition of diet they received: normolipidic diet, monounsaturated fatty acid diet with olive oil (olive oil diet) and saturated fatty acid diet with lard (lard diet) (Table 1). The diets administration was initiated after weaning and, after 60 days of diet, mice were infected with *T. cruzi*. The analyses were performed at the day 30 post infection (30 dpi), except in three independent experiments in which the survival rate and the blood parasitemia were followed by 60 days, the period when the number of parasites in the blood decreased.

**Table 1:** Diet composition (1,000 g).

**Ingredients (g)**	**Control diet**	**Hyperlipidic diet–Olive oil (DOO)**	**Hyperlipidic diet–Lard (DL)**
Corn starch	465.7	287.7	287.7
Casein	140.0	140.0	140.0
Dextrinized starch	155.0	155.0	155.0
Sucrose	100.0	100.0	100.0
Soy oil	40.0	40.0	40.0
Microcrystalline cellulose	50.0	50.0	50.0
Mineral mix AIN93M^a^	35.0	35.0	35.0
Vitamin mix AIN93M^b^	10.0	10.0	10.0
L-cysteine	1.8	1.8	1.8
Choline Bitartrate	2.5	2.5	2.5
Lard^c^	–	–	168.0
Cholesterol	–	–	10.0
Extra virgin olive oil^d^	–	178.0	–
Total caloric value/1,000 g	4020.0	4910.0	4910.0

a *Minerals (g/kg): CaCO_3_ 357.0/C_6_H_5_K_3_O7.H_2_O 28.0/KH_2_PO_4_ 250.0/NaCl 74.0/K_2_SO_4_ 46.6/MgO 24.0/C_6_H_5_ + 4.FexNyO_7_ 6.06/CO_3_Zn 1.65/MnCO_3_ 0.63/CuCO_3_ 0.3/KI 0.01/Na_2_Se 0.01025/(NH_4_) 6Mo_7_O_2_4.4H_2_O 0.00795/Na_2_SiO_3_.9H_2_O 1.45/KCr (SO_4_) 2.12 H_2_O 0.275/LiCl 0.0174/H_3_BO_3_ 0.0815/NaF 0.0635/NiCO3 0.0318/NH_4_VO_3_ 0.0066/C_12_H_2_O_11_ 209.806.*

b *Vitamins (mg/kg): Niacin 3.0/Calcium pantothenate 1.6/Pyridoxine 0.7/Thiamine 0.6/Riboflavin 0.6/Folic acid 0.2/Biotin 0.02/Vitamin E (500 IU/g) 15.0/Vitamin B12 (0.1%) 2.5/Vitamin A (500,000 IU/g) 0.8/Vitamin D3 (400,000 IU/g) 0.25/Vitamin K1/Dextrose Mix (10 mg/g) 7.50/Sucrose 967.23. Conversion factors: proteins 4 kcal/g, lipids 9 kcal/g, sugars 4 kcal/g*.

c *Fatty acid composition of lard (commercial name: Estrela–values referring to 100 g of the product): saturated 40.0/monounsaturated 44.76/polyunsaturated 15.42*.

d *Fatty acid composition of extra virgin olive oil (commercial name: Olivenza–values referring to 100 g of product): saturated 14.9/monounsaturated 75.6/polyunsaturated 9.5*.

### Food Intake and Lee's Index

The food intake was calculated by weighting the food provided every other day divided by the number of mice in the cage. The food intake (g) multiplied by the calorie provided, according to the offered diet) indicates the calorie intake. The Lee's index, indicator of obesity in rodents, proposed by Lee (14) and described by Bernardis and Petterson (15), was calculated by dividing the cubic root of body weight (g) by the naso-anal length (cm) and multiplied the result by 10.

### Biochemical Testing

Total cholesterol and triglycerides were determined using commercial kits from Bioclin® (Belo Horizonte, MG, Brazil) according to protocols available by the manufacturer.

### Liver Total Lipids Quantification

The total lipids in the liver were quantified according to Folch method. Briefly, 100 mg liver tissue was macerated in 400 mL of chloroform/methanol (2:1) and centrifuged at 3,000 g, for 10 min at 22°C. Following this, 800 mL of chloroform and 640 mL of NaCl (0.73%) were added to the supernatant, and samples were centrifuged at 3,000 rpm, for 10 min at 22°C. The lower phase was washed three times with 600 mL of Folch solution (a solution of distilled water containing 48% methanol, 3% chloroform, and 2% NaCl at 0.29%) and the extracted lipids were dried overnight at 50°C. The amount of lipid of each sample was calculated by the difference between the weight of samples before and after they were dried.

### Mice Infection, Parasitemia, Survival Rate, and Body Weight

Mice were inoculated intraperitoneally with 50 blood trypomastigote forms of the Colombian strain of *T. cruzi*, obtained after two consecutive passages of the strain in swiss mice. After the infection, the blood parasitemia levels were evaluated daily by counting the number of parasites in 5 ml tail-vein blood samples using an optical microscope. Mortality rate among the groups of animals was updated daily. In addition, the body weight was assessed daily by weighing the animals on a digital scale.

### DNA Extraction and Parasitism Analysis

The genomic DNA was extracted from 10 mg of heart or adipose tissue using the Wizard® SV Genomic DNA Purification System kit (Promega) according to the manufacturer's instructions. Real-time polymerase chain reaction (PCR) was performed to quantify the heart parasitism as previously described (16).

### Histology

To determine the number of cells infiltrated in the heart and epididymal adipose tissue, small pieces of the tissues were fixed in dimethyl sulfoxide (DMSO)-Methanol (1:5) for 30 days, dehydrated through successive incubations in crescent concentrations of ethanol, cleared in xylol and fixed in plastic paraffin (Paraplast^®^). The paraffin-fixed tissues were cut into sections with a size of 4 μm, stained with hematoxylin and eosin (HE) and the cell nuclei present in the fragments were quantified in 20 images (random fields). The images visualized by the 40X objective were scanned through the Leica DM 5000 B (Leica Application Suite, version 2.4.0R1) and processed through the Leica Qwin V3 image analyzer program.

### Quantitative Real-Time PCR

Total RNA from the heart and epididymal adipose tissue was extracted using the TRIzol reagent (Invitrogen) and the SV Total RNA Isolation System kit (Promega) according to the manufacturer's instructions. Complementary DNA was synthesized from 500 ng of RNA through a High Capacity cDNA Reverse Transcription kit (Applied Biosystems). Real-time PCR assays were performed in a StepOnePlus Real-Time PCR System (Applied Biosystems) using SYBR Green Mix reagents (Invitrogen). The mean cycle threshold (Ct) values from duplicate measurements were used to calculate the expression of the two target genes, which were normalized to the housekeeping genes *GAPDH* or *18S*. The sequences of all primers used are described in Table 2.

**Table 2:** Sequences of the primers used.

**Targets**	**Sequences**
*Tlr2*	Forward: *AAGTCTCCGGAATTATCAGTCC* Reverse: *TGATGGATGTCGCGGAT*
*Tlr9*	Forward: *GGACCTACAGCAGAATAGCTCA* Reverse: *AACTCGGGAACCAGACATG*
*T. cruzi*	Forward: *AAATAATGTACGGG(T/G)GAGATGCATGA* Reverse: *GGGTTCGATTGGGGTTGGTGT*
*18S*	Forward: *CCGCAGCTAGGAATAATGGAATA* Reverse: *GCCTCAGTTCCGAAAACCAA*
*Gapdh*	Forward: *GTGGAGTCATACTGGAACATGTAG* Reverse: *AATGGATGAAGGTCGGTGTG*

### Immunoassay

Levels of CCL2 were detected in the supernatant of the homogenized cardiac and adipose tissues. For sample preparation, 20 mg of heart and 40 mg of epididymal adipose tissues were macerated in 200 mL of phosphate buffered saline (PBS) and, after centrifugation at 1,500 g, for 10 min at 4°C, the supernatant was collected. Inflammatory mediators were measured by enzyme-linked immunosorbent assay (ELISA) using a specific kit (Peprotech^®^) and were performed according to the manufacturer's information. The absorbance values were measured using the eMax ELISA reader (Molecular Devices) at 450 nm.

### Catalase Activity Assay

Catalase activity was determined as described by Aebi (17) based on its ability to convert hydrogen peroxide (H_2_O_2_) into water and molecular oxygen. Briefly, 100 mg liver tissue was macerated in 1 mL of 0.1 M phosphate buffer, pH 7.2, centrifuged at 10,000 g, 10 min at 4°C. For the assay, 10 μL of the supernatant was added in 50 μL of K_2_HPO_4_, 40 μL of milli-Q water (Millipore, Bedford, MA) and 900 μL of 2.5 mmol/L H_2_O_2_. The enzyme activity was measured at 240 nm at 30 s, 2 and 3 min by decaying the absorbances. One-unit (U) of catalase is equivalent to the hydrolysis of 1 mmol of H_2_O_2_ per minute.

### Superoxide Dismutase Activity

Pyrogallol undergoes autoxidation producing the superoxide anion (O^−^). The SOD enzyme competes for the O^−^ by decreasing the 3-(4,5-Dimethylthiazol-2-yl) (MTT) reduction. For the assay, the supernatant of 100 mg of liver tissue was mixed with MTT and pyrogallol and incubated at 37°C for 5 min. The reaction was stopped with DMSO and the plate was read at 570 nm. The results were expressed as U of SOD per mg of protein in the supernatant of the liver tissue. One unit of SOD is defined as the amount of enzyme required for 50% inhibition of MTT reduction.

### Oxidized and Total Glutathione Quantification Assay

For the dosage of oxidized glutathione (GSSG), we used a standard solution of 10 mM oxidized glutathione for the samples and a standard solution of 50 mM oxidized glutathione in 5% sulfosalicylic acid (SSA) for the curve. The samples were obtained from the supernatant of 100 mg of liver homogenized in 1 mL of 5% sulfosalicylic acid buffer - SSA. Following this, 100 μL of the samples were pipetted in 50 mL tubes and pipetted between 0.5 and 2.0 μL of vinylpyridine and 1–5 μL of triethanolamine (TEA) was added to maintain the pH between 6.0 and 7.0. The tubes were filled with distilled water to a volume of 15 mL, homogenized and kept at room temperature for 1 h. After the incubation, 10 μL of samples were measured at 412 nm. The samples were incubated for additional 5 min at room temperature and, afterwards, 50 μL of nicotinamide adenine dinucleotide phosphate (NADPH) was added. The plate was read each 1 min during the 5 min incubation. Oxidized glutathione in the samples was calculated based on pre-defined concentrations for the calibration curve (p1: 0.25, p2: 0.125, p3: 0.062, p4: 0.0312, p5: 0.0156). For the measurement of total glutathione, 10 μL of samples were pipetted in a 96-well plate and immediately after the addition of 50 μL of NADPH, the absorbances were read each 1 min during the 5 min incubation. The values for reduced glutathione (GSH) were obtained from the difference between total and oxidized glutathiones.

### Statistical Analysis

Data were expressed as means ± SEM. Multiple groups were compared using one-way analysis of variance (ANOVA) followed by Tukey-Kramer *post-test*. The survival rate was compared by log-rank test (Mentel-Cox) and the student's *t*-test was used to compare differences among two experimental groups. All analyzes were performed using the Prism 8 software (GraphPad Software). Groups were considered statistically different when *p*-values < 0.05.

## Results

### Hyperlipidic Diet Alters Body and Biochemical Parameters in Mice

**Figure 1:**
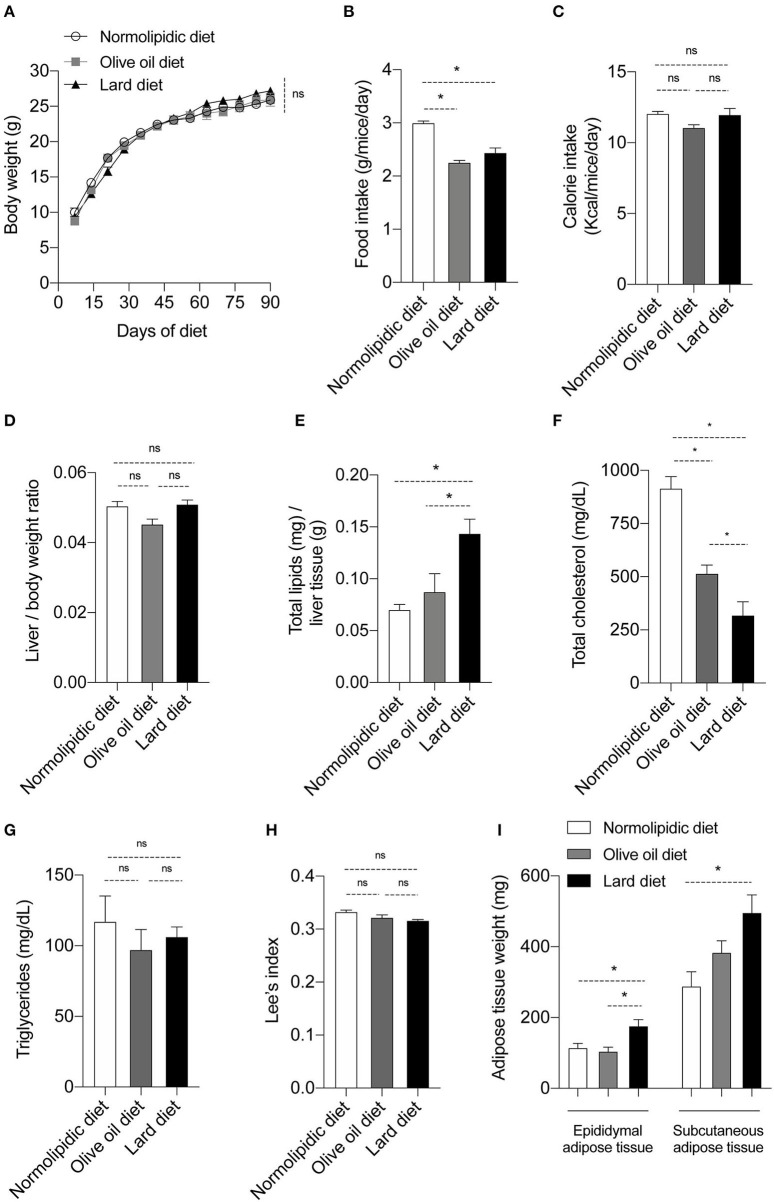
Body and biochemical parameters from mice fed with different types of hyperlipidic diets. Newly weaned C57BL/6 mice were fed with either a normolipidic diet (*n* = 8), monounsaturated fatty acid diet with olive oil (*n* = 8) or saturated fatty acid diet with lard (*n* = 8) and **(A)** the body weight was monitored weekly in a digital scale. The **(B)** food intake and the **(C)** calorie intake were calculated every other day. At the day 90th of diet, mice were euthanized and **(D)** the liver/body weight ratio, **(E)** liver total lipids levels, **(F)** plasma total cholesterol, **(G)** plasma triglycerides, **(H)** Lee's index and **(I)** weight of adipose tissue were evaluated. ns, not statistically different, *p < 0.05 by one-way ANOVA followed by Tukey *post-hoc* test.

Firstly, we evaluated if the intake of normal and hyperlipidic diets, and the calories provided by diets were similar among the groups. To address this point, newly weaned mice were fed with either a normolipidic diet, monounsaturated fatty acid diet (olive oil) or with a saturated fatty acid diet (lard) for 90 days. None of them modified mice's body weight (Figure 1A). Although mice fed with normolipidic diet consumed a higher quantity of food than groups fed with olive oil or lard diets (Figure 1B), the calorie intake was similar among all animals (Figure 1C). The effect of hyperlipidic diets on the biochemical and body parameters of mice were also evaluated. The liver/body weight ratio was not different among animals receiving distinct diets (Figure 1D) and mice fed a lard diet presented higher amount of liver lipids when compared to those fed with normolipidic and olive oil diets (Figure 1E). We observed a decreased plasma levels of total cholesterol in mice fed with olive oil and lard diets (Figure 1F) while triglycerides level was similar among all animals (Figure 1G). The Lee's index, an indicative degree of obesity in mice (18), was similar among all animals (Figure 1H), but the weight of the epididymal and subcutaneous adipose tissues was higher in association with the lard diet (Figure 1I).

### Diet Composition Interferes With the Heart and Adipose Tissue Parasitism After *T. cruzi* Infection

**Figure 2:**
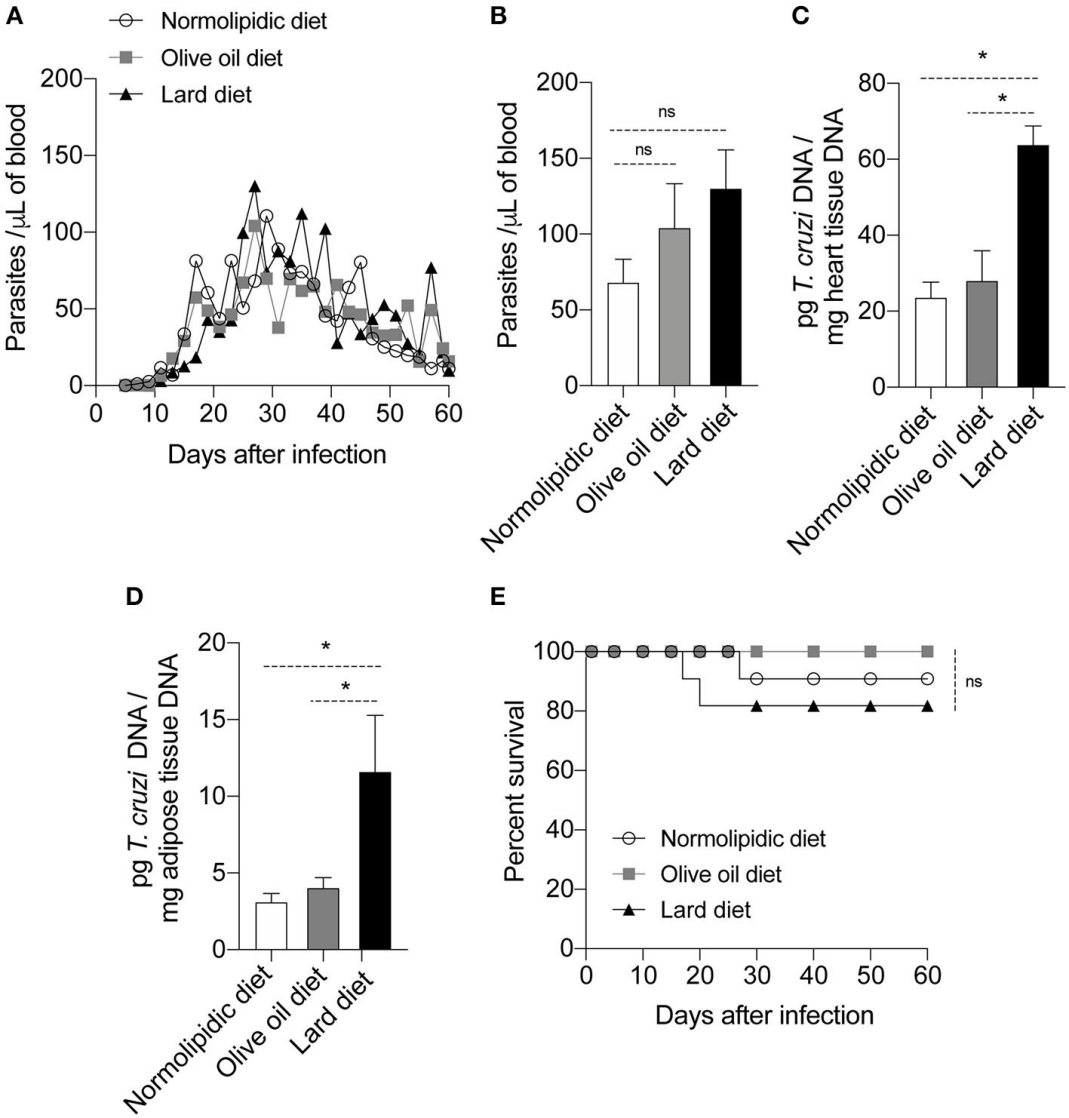
Parasitism and survival rate of mice fed with different types of hyperlipidic diets. Newly weaned C57BL/6 mice were fed with either a normolipidic diet (*n* = 8), monounsaturated fatty acid diet with olive oil (*n* = 8) or saturated fatty acid diet with lard (*n* = 8). At the day 60 of diet, mice were intraperitoneally infected with 50 blood trypomastigote forms of *T. cruzi* Colombian strain and **(A)** the blood parasite levels were monitored daily. **(B)** Number of parasites circulating in the mice blood at the day 27 after infection. At the day 30 post infection the DNA was extracted from **(C)** the heart and **(D)** adipose tissue and the amount of DNA from parasites were quantified in the tissues by real time PCR assay. **(E)** The survival rate of mice fed with normolipidic diet, olive oil diet or lard diet was monitored daily for 35 days. ns, not statistically different; *p < 0.05 by one-way ANOVA followed by Tukey *post-hoc* test.

To evaluate if the olive oil diet or lard diet would affect the response against the Colombian strain of *T. cruzi*, mice were infected after 60 days of diet. The number of parasites circulating in the mice blood was similar between the infected animals in all days evaluated (Figure 2A); the difference was not evident even during the peak of the parasitemia, at day 27 post infection (Figure 2B). Interestingly, at the day 30 post infection, mice fed with the lard diet presented higher parasitism in the heart (Figure 2C) and adipose tissue (Figure 2D) compared to the mice fed with normolipidic diet or olive oil diet. Despite the high number of parasites among the group fed with lard diet, the percentage of survival was not different between the groups that received the different types of diet (Figure 2E).

### Increased Amount of Olive Oil or Lard in the Diet Did Not Alter the Tissue Inflammation After *T. cruzi* Infection

**Figure 3:**
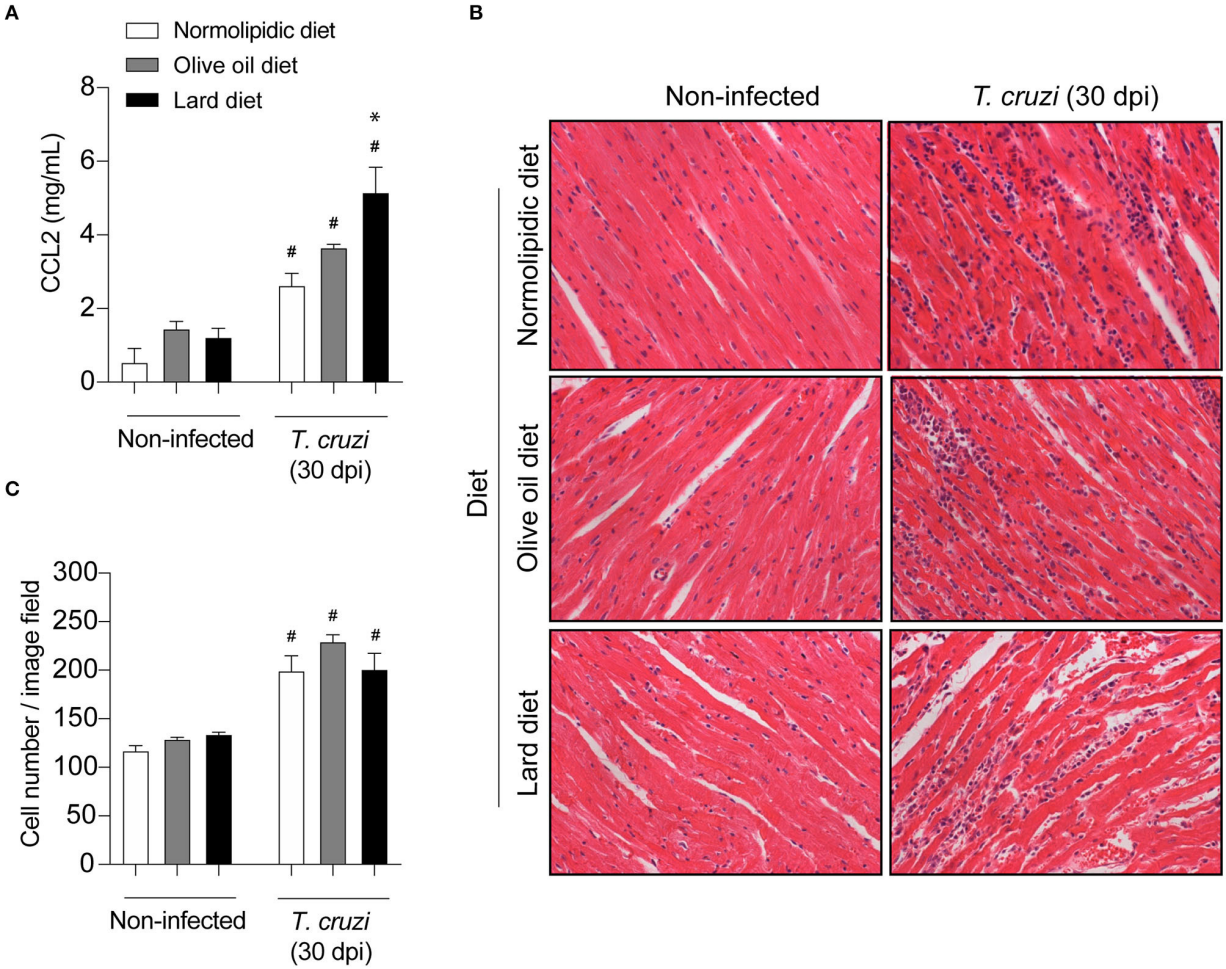
Cardiac inflammatory parameters of *T. cruzi* infected mice fed with different types of hyperlipidic diets. Newly weaned C57BL/6 mice were fed with either a normolipidic diet (*n* = 8), monounsaturated fatty acid diet with olive oil (*n* = 8) or saturated fatty acid diet with lard (*n* = 8). At the day 60 of diet, mice were intraperitoneally infected with 50 blood trypomastigote forms of *T. cruzi* Colombian strain. After 30 dpi **(A)** the concentration of CCL2 was assessed in the heart tissue by ELISA assay. **(B)** The heart tissue was fixed, processed, blocked in paraffin, cut, and stained with hematoxylin and eosin and **(C)** the number of cells in the tissue was quantified. ^#^*p* < 0.05 compared with the non-infected group and *p < 0.05 compared with infected normolipidic diet group by one-way ANOVA followed by Tukey *post-hoc* test.

**Figure 4:**
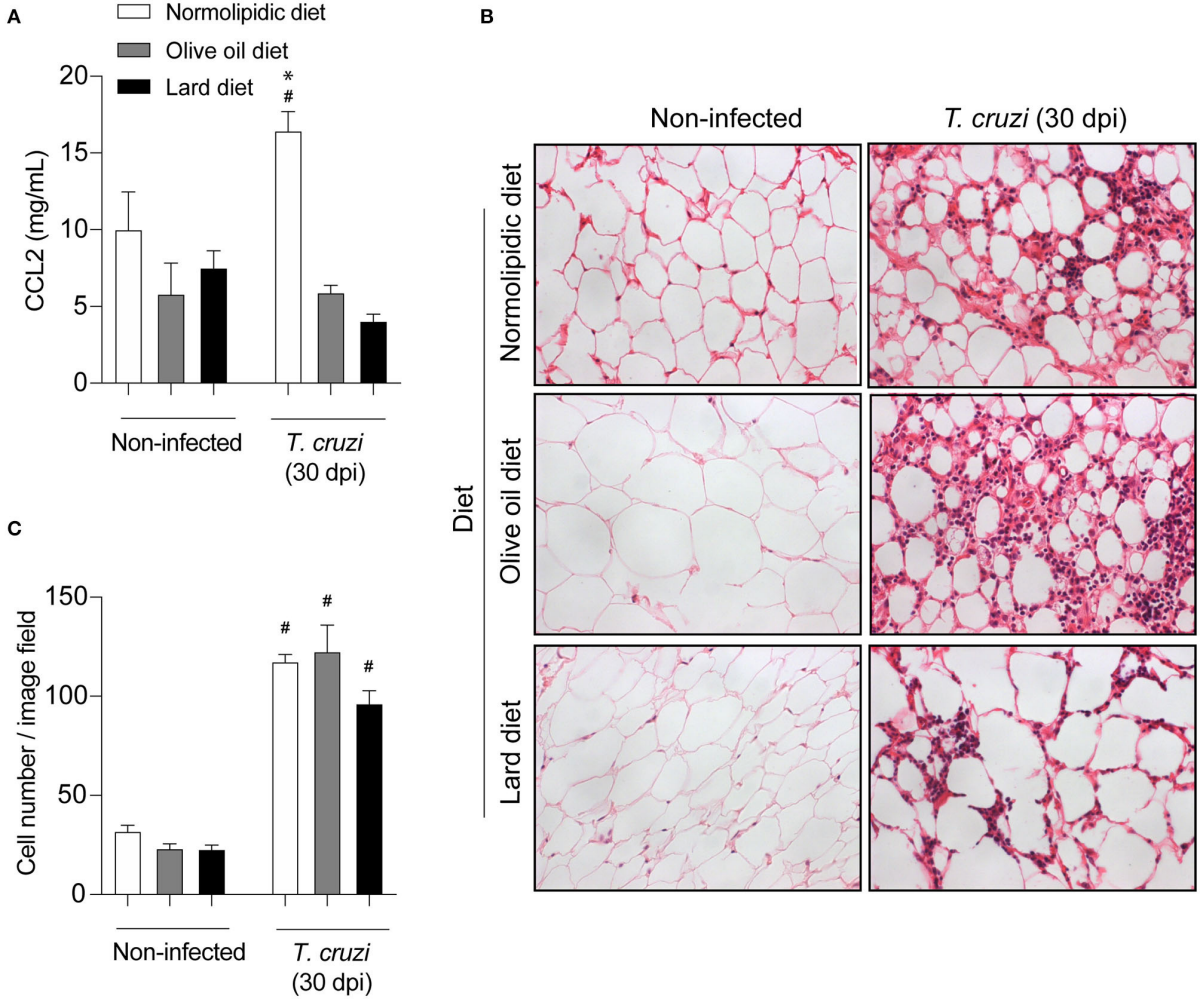
Adipose tissue inflammatory parameters of *T. cruzi* infected mice fed with different types of hyperlipidic diets. Newly weaned C57BL/6 mice were fed with either a normolipidic diet (*n* = 8), monounsaturated fatty acid diet with olive oil (*n* = 8) or saturated fatty acid diet with lard (*n* = 8). At the day 60 of diet, mice were intraperitoneally infected with 50 blood trypomastigote forms of *T. cruzi* Colombian strain. After 30 dpi **(A)** the concentration of CCL2 was assessed in the adipose tissue by ELISA assay. **(B)** The adipose tissue was fixed, processed, blocked in paraffin, cut, and stained with hematoxylin and eosin and **(C)** the number of cells in the tissue was quantified. ^#^*p* < 0.05 compared with the non-infected group and *p < 0.05 compared with infected olive oil and lard diets by one-way ANOVA followed by Tukey *post-hoc* test.

Since mice fed with lard diets presented with high tissue parasitism, we investigated whether the diets interfere with the migration of inflammatory cells to the heart and adipose tissue following infection. For this purpose, after 60 days of either normolipidic, olive oil or lard diets followed by 30 dpi with *T. cruzi* Colombian strain, the production of the chemokine CCL2 and the tissue inflammation were evaluated. The infection increased the CCL2 production in the heart tissue (Figure 3A) and the number of inflammatory cells infiltrating the tissue (Figures 3B,C) independently of the diet administered. Although the lard diet increases the heart CCL2 production after infection compared with infected normolipidic group, the number of inflammatory cells in the tissue is similar among the infected groups independent of the dietary type (Figures 3B,C). Mice fed with the normolipidic diet presented with an increased production of CCL2 in adipose tissue after infection compared to all other dietary group (Figure 4A) despite this, the tissue inflammation increased similarly for all groups (Figures 4A–C).

### Hyperlipidic Diet Rich in Olive Oil Increases Toll-Like Receptors Expression in the Cardiac Tissue After *T. cruzi* Infection

**Figure 5:**
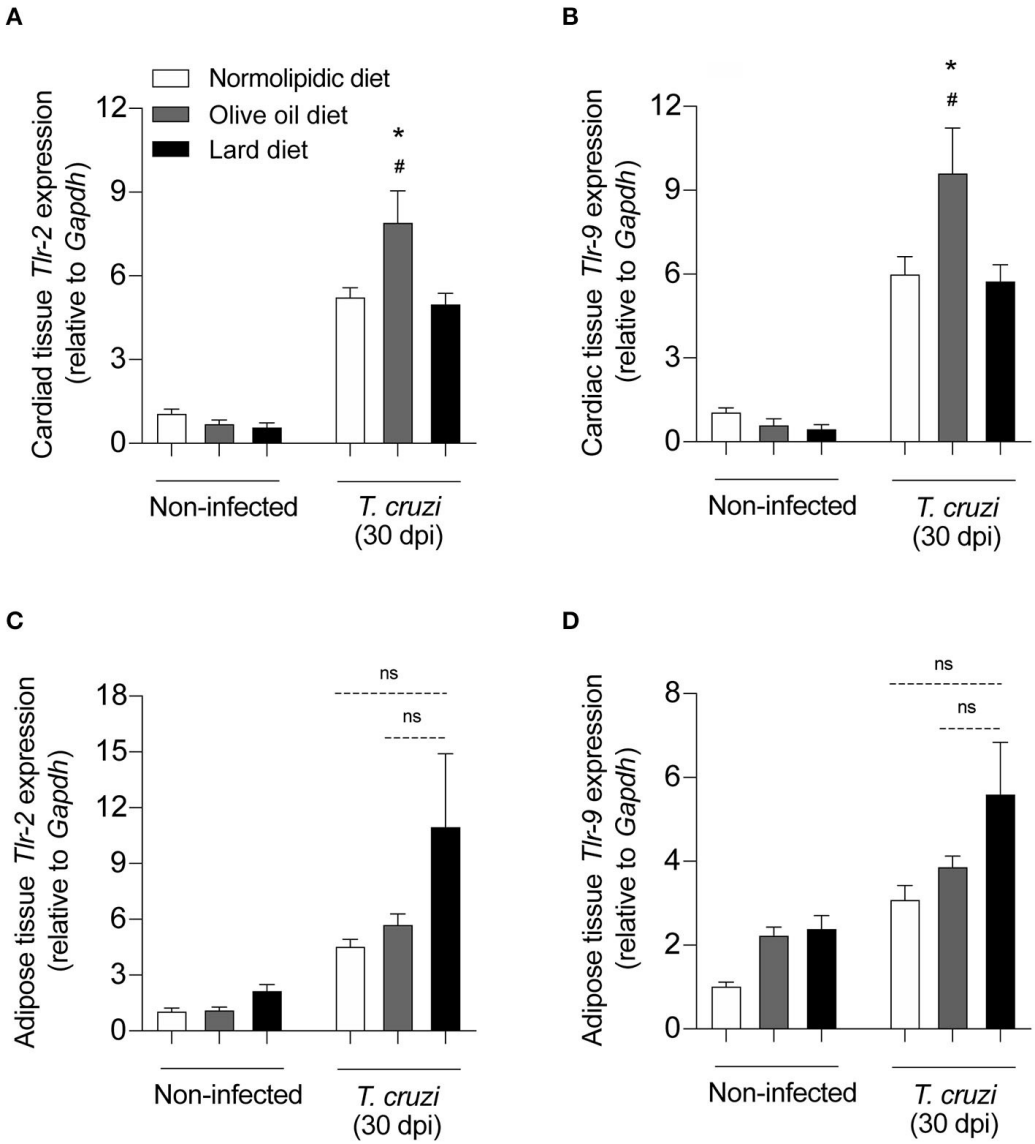
TLRs expression on the heart and adipose tissue from *T. cruzi* infected mice fed with different types of hyperlipidic diets. Newly weaned C57BL/6 mice were fed with either a normolipidic diet (*n* = 5), monounsaturated fatty acid diet with olive oil (*n* = 5) or saturated fatty acid diet with lard (*n* = 5). At the day 60 of diet, mice were intraperitoneally infected with 50 blood trypomastigote forms of *T. cruzi* Colombian strain. After 30 dpi the mRNA expression levels of *Tlr2* and *Tlr9* were measured in the heart **(A,B)** and adipose tissue **(C,D)** by real time quantitative PCR. *Gapdh* and *18S* were used as housekeeping genes for the heart and adipose tissues, respectively. ns, not statistically different. ^#^*p* < 0.05 compared with the non-infected group and *p < 0.05 compared with infected group by one-way ANOVA followed by Tukey *post-hoc* test.

Since TLRs are particularly important for parasite recognition by immune cells, we investigated whether the hyperlipidic diets could interfere with the TLR expression in the cardiac and adipose tissue cells after *T. cruzi* infection. After receiving either normolipidic, olive oil or lard diets for 60 days, mice were infected by Colombian strain of *T. cruzi* and the mRNA expression levels of *Tlr2* and *Tlr9* were evaluated in the cardiac and adipose tissues of infected and non-infected mice. Infected mice fed with olive oil diet presented higher *Tlr2* and *Tlr9* expression in the heart (Figures 5A,B) while those fed with lard diet presented higher *Tlr2* and *Tlr9* expression in the adipose tissue (Figures 5C,D).

### Hyperlipidic Diet Alters the Redox Status After *T. cruzi* Infection

**Figure 6:**
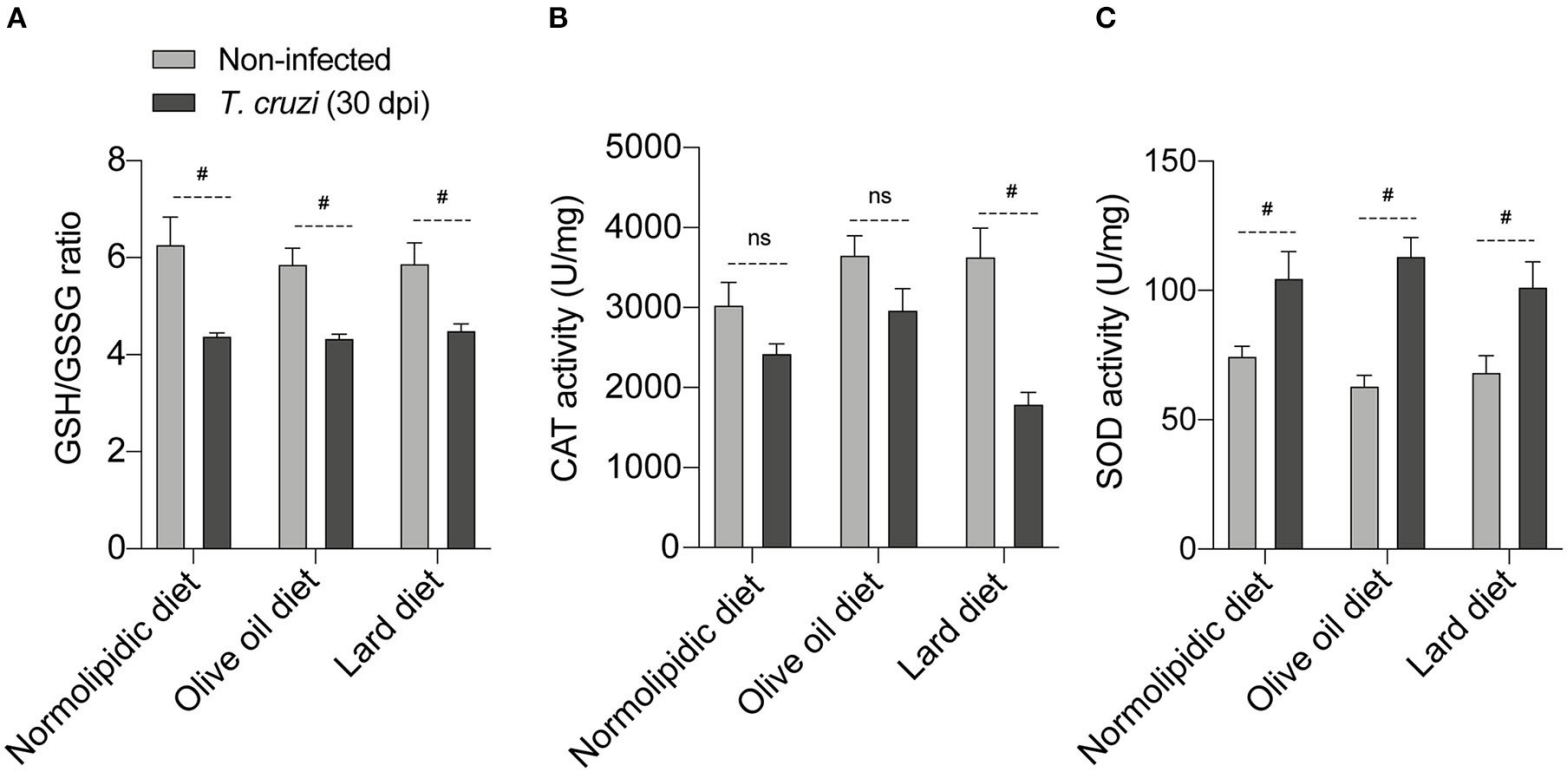
Redox status of *T. cruzi* infected mice fed with different types of hyperlipidic diets. Newly weaned C57BL/6 mice were fed with either a normolipidic diet (*n* = 5), monounsaturated fatty acid diet with olive oil (*n* = 5) or saturated fatty acid diet with lard (*n* = 5). At the day 60 of diet, mice were intraperitoneally infected with 50 blood trypomastigote forms of *T. cruzi* Colombian strain. After 30 dpi **(A)** the GSH/GSSG ratio, **(B)** CAT activity and **(C)** SOD activity were measured in the supernatant of a macerated piece of liver. GSH, reduced glutathione; GSSH, oxidized glutathione; CAT, catalase; SOD, superoxide dismutase; ns, not statistically different; ^#^*p* < 0.05 by student *t*-test.

The *T. cruzi* infection-caused oxidative stress, resulting in the accumulation of free radicals, which altered the expression and/or activity of antioxidant enzymes such as oxidized glutathione (GSSG), catalase (CAT), and superoxide dismutase (SOD). Activities of GSSG, CAT and SOD were evaluated in the liver of mice fed with either a control, olive oil, or lard diet for 60 days, followed by 30 dpi with *T. cruzi*. The infection significantly downregulated the GSH/GSSG ratio in a diet-independent manner (Figure 6A). In response to the parasite-induced oxidative stress, we observed a slight decreased in the CAT activity in mice under normolipidic or olive oil diets, while infected mice under lard diet presented with intense reduction of catalase activity (Figure 6B). In addition, SOD is important for the protection of cells from oxidative insults, and we observed increases in SOD activity after 30 dpi. This increase was independent of the dietary type (Figure 6C).

## Discussion

The ingested lipids in foods are essential elements that impacts the inflammatory profile or alter the redox status caused by the inflammation (19). Diet exerts an important role during the inflammatory processes, with some nutritional studies demonstrating that ample and restricted consumption of monounsaturated and saturated lipids, respectively, decreases the expression of low-grade inflammatory markers (20, 21). The effects of a high fat diet in inflammation caused by *T. cruzi* are partially known (22, 23), but the effects of different types of lipids remain poorly studied.

Although high consumption of saturated fatty acids is frequently associated with weight gain and obesity in humans and experimental models (24, 25), we did not observe alterations in the body or liver weights in our lard group compared with olive oil and normolipidic dietary groups. Our results were consistent with other studies that have shown that few weeks under high fat diet rich in saturated lipids do not increase the body weight in experimental models (26, 27). Interestingly, Marques and collaborators showed that the diet-induced weight gain depends on the strain of rats and some strains require longer exposure to the diets to gain weight (28). Although the weight was comparable across dietary groups, the lard diet increased the liver lipid deposition. This could indicate hepatic steatosis commonly observed after hyperlipidic diets (11). In addition, there are unclear points that limit the interpretation of rodent lipid metabolism with the same optical prism used to humans (29). Of note, the majority of human plasma cholesterol is transported as low-density lipoproteins (LDL), but mice do not have cholesteryl ester transfer protein, and for this reason, significant amounts of cholesterol are identified as high-density lipoproteins (HDL) in these animals. Another point, when animals have a high consumption of carbohydrates (e.g., normolipidic diet - AIN-93M), they can modify cholesterol metabolism increasing it in the blood (30), even under a low-lipid diet, as we have shown in this current study.

Hyperlipidic diets discussed here present equal energy density, however, the lard diet is the more efficient in transforming calories into adipose tissue assuming the normolipidic diet as reference. No change was observed in the body composition of animals under the effect of the different diets, according to Lee's index, however, the increased epididymal adipose tissue related to a lard diet may be favoring parasite interaction with host cells due to the presence of saturated fatty acids and the increased cell internalization of cholesterol, as previously demonstrated (7, 31–34). Then, considering the cholesterol analogs for the parasite invasion and replication and, considering both olive oil and lard diets have similar total lipid percentage, we supplemented the lard diet with 1% cholesterol attempting to understand how it could be favorable or not to the *T. cruzi*.

Previously published results have showed that the *T. cruzi* infects and proliferates within brown and white adipose tissue (32, 35). In accordance with this, we showed that, although the blood parasitemia was the same in mice from the hyperlipidic and normolipidic dietary groups, mice under lard diet showed increased parasitism in both the adipose and cardiac tissues. Despite high tissue parasitism, the survival rate was the same among all dietary groups. In contrast, using the Brazil strain of *T. cruzi*, Nagajyothi et al. demonstrated that a high fat diet increases survival rate in infected mice (7). Of note, studies involving *T. cruzi* must pay attention to the strain used. A limitation of our study was that, given that mice fed a normolipidic diet survived the infection with the Colombian strain, we were not able to observe higher survival rates in infected animals from the other dietary groups.

Chemokine production and tissue recruitment of inflammatory cells in *T. cruzi* infected animals are essential for proper parasite control (36). In fact, our findings demonstrated an increase in heart CCL2 expression and the number of inflammatory cells in the heart and adipose tissues following infection in all dietary groups. Although an increase in CCL2-producing macrophages is expected in the adipose tissues of infected mice (37), mice from lard dietary group presented with adipose tissue inflammation, independent of CCL2 production. Worth highlighting that caloric restriction has been shown to reduces migration of peripheral inflammatory monocytes from the bone marrow to the circulation and tissues by interference on the CCL2 production in a mechanism dependent on the peroxisome proliferator-activator receptor alpha and the activated protein kinase (38). In our study, animals fed with normolipidic performed higher ingestion of food in relation to those feed with olive oil or lard diets, however without differences in the calorie intake. In addition, the lard diet promoted a higher parasites load in both evaluated sites evaluated with the distinct pattern of CCL2 expression, after 48 h of the peak of parasitemia concerning Colombian strain of *T. cruzi*. At this representative moment of the experimental *T. cruzi* infection, no difference in inflammatory infiltration was detected through histological sections, but based on our previous experience, higher expression of CCL2 represents a higher release of this chemokine, an increasing of leukocyte recruitment, higher cardiac tissue damage and mortality in those infected animals (39).

Also essential to parasite control by immune cells is the recognition of *T. cruzi* structures by cellular receptors. The importance of TLR-2 and TLR-9 during the *T. cruzi* infection was previously reported (40). Our results demonstrated an overexpression of *Tlr2* and *Tlr9* mRNA in adipose tissue and in cardiac tissue driven by lard and olive oil diets, respectively. Recent evidence suggest that saturated fatty acids can promote TLR-2 and TLR-4 activation while polyunsaturated fatty acids can inhibit these receptors (41). In parallel, *T. cruzi*-glycosylphosphatidylinositol membrane anchor is recognized by both, TLR-2 and TLR-9, and exert a role in the prognosis of asymptomatic and cardiac clinical forms of Chagas disease (42). In note, *T. cruzi* infection increases *Tlr2* and *Tlr9* expression in brown and white adipose tissues, respectively, as well as in other immune cells (43), and, together, they act in the balance between modulatory and inflammatory responses. This duality observed in the expression of *Tlr2* and *Tlr9* under high-fat diets effects might interfere in the parasite-host interaction and the immunopathogenesis since the deficiency of these receptors or of the Myd88 promotes susceptibility in infected mice (40) or the activation of *TLR2* and NF-Kb triggers cardiomyocyte hypertrophy (44, 45). Remarkably, the toll-like receptors act as a bridge between diet-induced endocrine and immune response, but further investigations are necessary to better understand this network.

Finally, both lipid-rich diets and *T. cruzi* infection cause oxidative stress (46, 47), and consequently, require the regulation of the antioxidant levels to reduce the high free radicals levels. The parasite decreases the GSH/GSSG ratio and CAT activity and increases SOD activity increasing the oxidative stress (48, 49). Interestingly, and in accordance with previous published data (23), our mice fed with saturated lipid-rich diet showed decreased CAT activity compared with mice from the normolipidic and unsaturated lipid-rich dietary groups. The antioxidant CAT rescues the cardiac dysfunction induced by high fat diets in mice (50). This suggests that the reduced CAT levels during infection may contribute to parasite-caused cardiac pathogenesis.

In summary, our findings provided evidence that diets rich insaturated lipids (e.g., lard diet) promote growth of *T. cruzi* in tissues following infection, and decrease the liver antioxidant production, contributing to the tissue damage.

## Data Availability Statement

The raw data supporting the conclusions of this article will be made available by the authors, without undue reservation.

## Ethics Statement

The animal study was reviewed and approved by Animal Research Ethics Committee of the Federal University of Ouro Preto (CEUA-UFOP), under protocol number 36/2015.

## Author Contributions

DMSS, MCS, and AT: design, writing, and final content. DMSS, SEBF, APJM, CMM, KPL, NCNP, PMA, DCC, and GPC: performed the experiments. DMSS, MCS, and KMCP: data analysis. JSS and AT: funding acquisition. All authors have read and approved the final version of this manuscript.

## Conflict of Interest

The authors declare that the research was conducted in the absence of any commercial or financial relationships that could be construed as a potential conflict of interest.
